# Spinal leptomeningeal disease in patients with solid tumors: A single-center study on clinical presentation, diagnosis, treatment, and outcomes

**DOI:** 10.1016/j.bas.2026.106660

**Published:** 2026-08-29

**Authors:** Zoe Shaked, Chiara Eitner, Claudius Jelgersma, Anton Früh, Ran Xu, Helena Radbruch, Jawed Nawabi, Felix Ehret, Güliz Acker, Daniel Zips, Lars Wessels, Ulrich Keilholz, Julia Onken, Martin Misch, Peter Vajkoczy, David Wasilewski

**Affiliations:** a Charité – Universitätsmedizin Berlin, Corporate Member of Freie Universität Berlin and Humboldt-Universität zu Berlin, Department of Neurosurgery, Berlin, Germany; b German Center for Cardiovascular Research (DZKK), Berlin, Germany; c Charité – Universitätsmedizin Berlin, Corporate Member of Freie Universität Berlin and Humboldt-Universität zu Berlin, Department of Neuropathology, Berlin, Germany; d Charité – Universitätsmedizin Berlin, Corporate Member of Freie Universität Berlin and Humboldt-Universität zu Berlin, Department of Neuroradiology, Berlin, Germany; e Berlin Institute of Health at Charité, Universitätsmedizin Berlin, Berlin Institute of Health (BIH) Charité, Charitéplatz 1, Berlin, 10117, Germany; f Charité – Universitätsmedizin Berlin, Corporate Member of Freie Universität Berlin and Humboldt-Universität zu Berlin, Department of Radiation Oncology, Berlin, Germany; g German Cancer Consortium (DKTK), partner site Berlin, a partnership between German Cancer Research Center (DKFZ)and Charité - Universitätsmedizin Berlin, Berlin, Germany; h Charité – Universitätsmedizin Berlin, Corporate Member of Freie Universität Berlin and Humboldt-Universität zu Berlin, Charité Comprehensive Cancer Center, Berlin, Germany; i Charité - Universitätsmedizin Berlin, Corporate Member of Freie Universität Berlin und Humboldt-Universität zu Berlin, Department of Hematology, Oncology and Cancer Immunology, Berlin, Germany

**Keywords:** Leptomeningeal disease, Spinal leptomeningeal disease, Vertebral metastasis, Intraspinal metastasis, Survival

## Abstract

**Introduction:**

Despite affecting 5-15% of metastatic cancer patients, leptomeningeal disease (LMD), and spinal LMD (sLMD) in particular, remain largely understudied.

**Research question:**

This study evaluates the clinical presentation, diagnosis, treatment, and outcomes of sLMD.

**Material and methods:**

We retrospectively analyzed sLMD patients treated at a tertiary center (2011–2024) who underwent cranial and spinal imaging within 30 days of LMD diagnosis. Survival analysis was performed using Kaplan-Meier curves, with a multivariable Cox regression model being employed to identify independent prognostic factors using a stepwise backwards approach.

**Results:**

Fifty-two patients (median age: 57.9 years, 63.5% female) were included. Breast cancer (30.8%) and NSCLC (25.0%) were the most common primary malignancies. Median time from primary disease diagnosis to LMD diagnosis was 30.2 months [95% CI: 18.9-42.6]. LMD was diagnosed in 51.9% of cases through imaging and in 48.1% through imaging and CSF cytology. At diagnosis, 92.3% had concurrent cranial and spinal LMD, 67.3% brain metastases, 50.0% vertebral metastases, and 19.2% intraspinal metastases. The most common symptoms were clinical decline (80.8%) and headaches (59.6%). Spine-related symptoms included radiculopathy (26.9%) and paraplegia (23.1%). Median overall survival was 2.7 months [95% CI: 2.0-4.1]. Independent prognostic factors for improved survival included systemic therapy following LMD diagnosis (HR = 0.40, p < 0.01), absence of cranial nerve deficits (HR = 0.45, p < 0.05) and absence of gait imbalance (HR = 0.50, p < 0.05) at LMD diagnosis.

**Discussion and conclusion:**

By highlighting the clinical challenges, heterogenous treatment regimens and poor prognosis associated with sLMD, this study serves as a foundation for further research aimed at improving management strategies and outcomes for this patient cohort.

## Highlights

• Despite advances in cancer care, the prognosis of spinal LMD remains dismal.
• Neither vertebral nor intraspinal metastases were associated with survival in sLMD.
• Age and KPS were not associated with survival, warranting further exploration of their role in therapy decisions in sLMD.
• Therapy was highly variable, reflecting the lack of standardized recommendations.
• Systemic therapy post-LMD diagnosis was associated with better survival.

## 1 Introduction

Leptomeningeal disease (LMD), also known as leptomeningeal metastasis and neoplastic meningitis, is a devastating sequela of metastatic disease characterized by the dissemination of cancer cells to the cerebrospinal fluid (CSF) and the leptomeninges. The route by which tumor cells spread to the CSF and meninges is thought to depend on the locationof the primary tumor (i.e., intracranial or extracranial) as well as its histopathology. Proposed routes include hematogenous dissemination via systemic arterial and venous circulation, perineural spread along cranial, spinal, and peripheral nerves, spread from the bone marrow through vascular channels, meningeal lymphatic invasion, and spread from adjacent metastatic lesions (Freret and Boire, 2024; Remsik and Boire, 2024; Yue et al., 2025; Ozair et al., 2024; Sener et al., 2025). LMD historically affected approximately 5-15% of patients with solid tumors during the course of their disease, with the incidence increasing to around 20% in recent years. This rise in incidence is attributable to advancements in local and systemic therapies that have prolonged the survival of patients with systemic cancer, including those with parenchymal brain metastases (BrMs). Hence, survival is likely to continue to increase in the future, further exacerbating the burden of LMD (Yue et al., 2025; Ozair et al., 2024; Le Rhun and Galanis, 2016; Le Rhun et al., 2017a; Le Rhun et al., 2017b; Taillibert and Chamberlain, 2018; Bonig et al., 2019; Ferguson et al., 2021; Wilcox et al., 2024; Thakkar et al., 2020; Nayar et al., 2017; Roy-O'Reilly et al., 2023; Pellerino et al., 2021; Le Rhun et al., 2019; Palmisciano et al., 2022). Similarly to parenchymal BrMs, the underlying primary entities most frequently associated with LMD include breast cancer**,** lung cancer, and malignant melanoma (Yue et al., 2025; Sener et al., 2025; Le Rhun and Galanis, 2016; Le Rhun et al., 2017a; Le Rhun et al., 2017b; Taillibert and Chamberlain, 2018; Ferguson et al., 2021; Wilcox et al., 2024; Thakkar et al., 2020; Nayar et al., 2017; Roy-O'Reilly et al., 2023; Pellerino et al., 2021; Le Rhun et al., 2019; Palmisciano et al., 2022). In standard clinical practice, LMD is typically diagnosed using CSF cytology or magnetic resonance imaging (MRI), preferably of the entire craniospinal axis (Yue et al., 2025; Ozair et al., 2024; Sener et al., 2025; Le Rhun and Galanis, 2016; Le Rhun et al., 2017a; Le Rhun et al., 2017b; Taillibert and Chamberlain, 2018; Bonig et al., 2019; Ferguson et al., 2021; Wilcox et al., 2024; Thakkar et al., 2020; Nayar et al., 2017; Roy-O'Reilly et al., 2023; Pellerino et al., 2021; Le Rhun et al., 2019; Palmisciano et al., 2022; Turner et al., 2020; Nguyen et al., 2021). Based on the disease's radiological appearance, the European Association of Neuro-Oncology (EANO) and European Society for Medical Oncology (ESMO) guideline distinguish between linear “sugar-coating” or classic LMD and nodular LMD, consisting of extra-axial nodular contrast-enhancing lesions on MRI which have close contact with meninges or ependyma (Ozair et al., 2024; Le Rhun et al., 2017b; Turner et al., 2020). Current data on the prognostic value of underlying LMD patterns are conflicting (Nguyen et al., 2020, 2021; Dankner et al., 2021). Treatment strategies depend on MRI subtype and various other factors such as the clinical performance status, underlying primary tumor type, intracranial disease status, extracranial disease status and previous treatments received. Therapies of LMD are, however, even in the case of aggressive multimodal treatment, largely palliative, and their efficacy is limited (Yue et al., 2025; Ozair et al., 2024; Sener et al., 2025; Le Rhun and Galanis, 2016; Le Rhun et al., 2017b; Taillibert and Chamberlain, 2018; Ferguson et al., 2021; Wilcox et al., 2024; Thakkar et al., 2020; Nayar et al., 2017; Roy-O'Reilly et al., 2023; Pellerino et al., 2021; Le Rhun et al., 2019; Palmisciano et al., 2022; Nguyen et al., 2021; Dankner et al., 2021; Mack et al., 2016; Wang et al., 2018; Wang et al., 2025; Smith et al., 2024; Ochoa-Lantigua et al., 2026; Sener et al., 2021). Hence, despite improved control of extracranial metastases (EcM) and parenchymal BrMs, outcomes for LMD remain dismal, with overall survival (OS) often limited to a few months (Yue et al., 2025; Ozair et al., 2024; Sener et al., 2025; Le Rhun and Galanis, 2016; Le Rhun et al., 2017a; Le Rhun et al., 2017b; Taillibert and Chamberlain, 2018; Bonig et al., 2019; Ferguson et al., 2021; Wilcox et al., 2024; Thakkar et al., 2020; Nayar et al., 2017; Roy-O'Reilly et al., 2023; Pellerino et al., 2021; Le Rhun et al., 2019; Palmisciano et al., 2022; Nguyen et al., 2021; Dankner et al., 2021; Nguyen et al., 2020; Mack et al., 2016; Wang et al., 2018; Wang et al., 2025; Smith et al., 2024; Ochoa-Lantigua et al., 2026; Sener et al., 2021; Yang et al., 2022). Most studies on LMD focus on the disease's cranial manifestation, while sLMD remains underinvestigated. Consequently, the clinical course of sLMD, optimal treatment regimens, and significant prognostic factors are not well established, warranting further investigation. Moreover, while the presence of vertebral and intraspinal metastases often correlates with worse prognosis, the implications of these manifestations for the pathogenesis of sLMD and their prognostic value remain poorly understood (Kokkoris, 1983). This single-center study aims to provide a detailed characterization of sLMD patients, including demographic features, clinical presentation, diagnosis, treatment, and outcomes.

## 2 Materials and methods

### 2.1 Patient cohort and data collection

Adult patients with a diagnosis of a solid, non-nervous-system malignancy and a radiological and/or neuropathological confirmation of LMD treated at one of the three sites of a large tertiary center between July 2011 and August 2024 were identified through our institutional electronic medical records. As shown in the CONSORT diagram (Fig. 1), patients with sLMD, who have undergone cranial and spinal imaging within 30 days of the initial LMD diagnosis (i.e., first evidence of LMD, detected either by CSF cytology or imaging), were identified and included in this cohort study (n = 52). Patients were followed until death; patients alive or lost to follow-up were censored at their last known clinical contact. Two patients were lost to follow-up as their place of primary residence is abroad; they were censored at their last clinical contact. Due to the study's retrospective nature, which entails no additional risk to the participating patients, informed consent was institutionally waived.

**Fig. 1:**
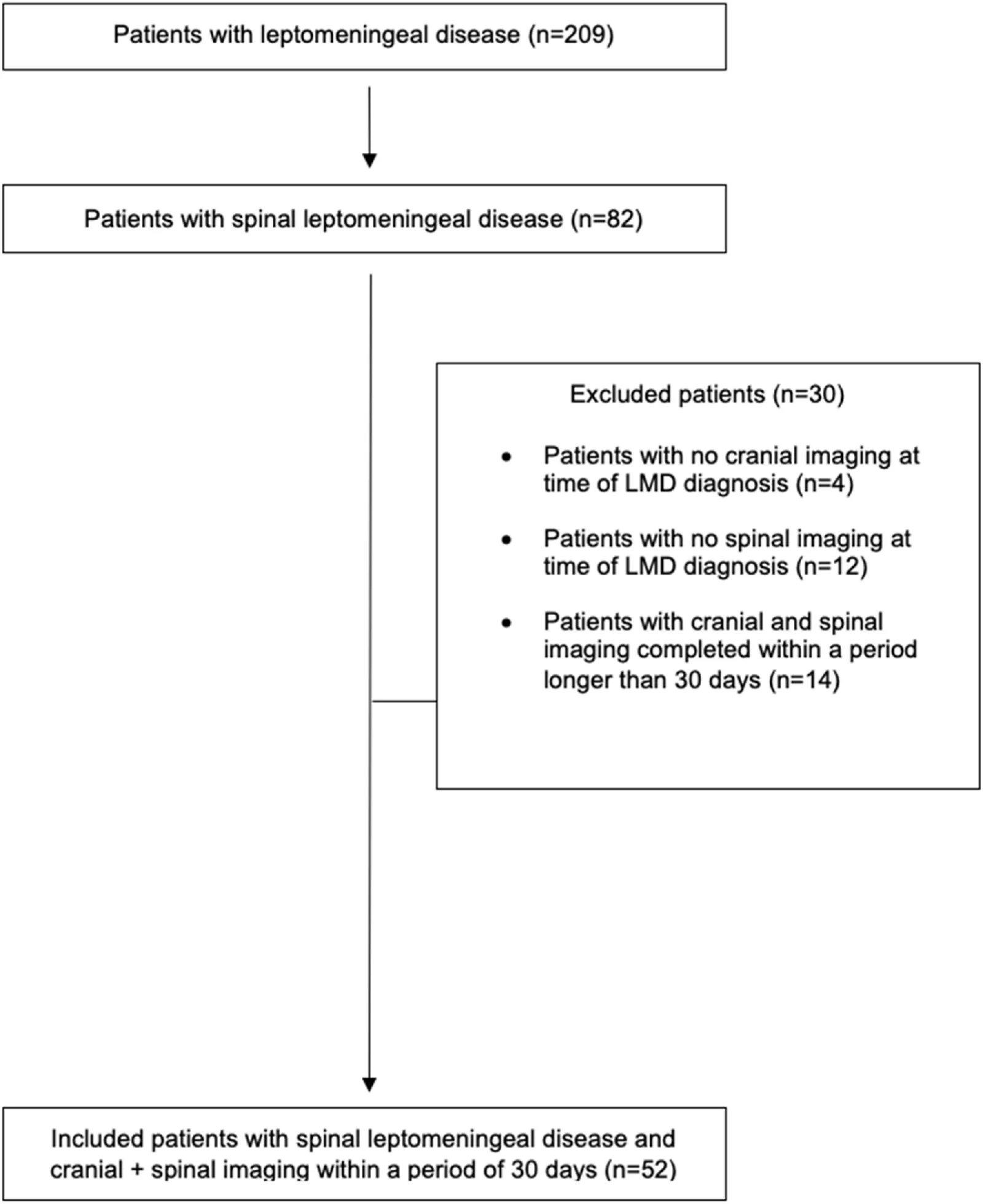
CONSORT diagram. CONSORT diagram demonstrating patient flow. Between July 2011 and August 2024, 209 patients with solid tumors were treated at our neurosurgical center for LMD, of which 82 patients had spinal LMD. Fifty-two patients fulfilled our inclusion criteria and were included in this study. Two patients who did not receive a dedicated complete spinal MRI but rather a cervical spine MRI and an MRI of the upper extremity on which the spine was also visible, respectively, were also included in our cohort.

### 2.2 Clinical and radiological variables

Clinical variables collected include demographic data (e.g., age, sex, and underlying tumor entity), data related to the patients’ course of disease (e.g., date of LMD diagnosis, presence of extra- and parenchymal intracranial metastases), radiological and pathological findings (e.g., cranial and spinal MRI studies at diagnosis, presence of tumor cells in CSF analysis), and treatment-related data (Table 1, Table 2, Table 3). Apart from the treatment-related data included in other studies pertaining to LMD, such as information regarding radiotherapy (RTx), microsurgical metastasis resections, and systemic therapy modalities, we also collected data on spinal interventions. In our study, spinal interventions were defined as any surgical procedure involving the vertebral column or spinal cord, performed for either diagnostic (e.g., biopsy of intraspinal metastasis) or therapeutic purposes (e.g., surgical treatment of concomitant spinal metastasis or their sequalae). Based on radiological findings, LMD was described as either classical or nodular according to the classification by Turner et al. (2020) and classified into MRI subtypes following the EANO ESMO recommendation (Le Rhun et al., 2017b). Intracranial and extracranial (non-central nervous system) metastases were assessed through MR and CT imaging, respectively. For the response and progression assessment, we used the response and assessment in neuro-oncology brain metastases (RANO-BM) criteria and the response evaluation criteria in solid tumors (RECIST) criteria (version 1.1); tumor activity was classified as complete response (CR), partial response (PR), stable disease (SD) or progressive disease (PD) (Lin et al., 2015; Eisenhauer et al., 2009). Metastases of the spine or spinal cord were assessed using MRI or CT imaging. The extent to which vertebral metastases infiltrate the epidural space at LMD diagnosis was evaluated using axial MR or CT images taken within 30 days of LMD diagnosis (if available) and classified using the Epidural Spinal Cord Compression (ESCC) scale (also known as Bilsky scale), which is instrumental in evaluating vertebral and epidural disease, and includes different degrees of spinal canal stenosis and spinal cord compression (bone-only disease, epidural impingement without spinal cord compression (without and with abutment), spinal cord compression with CSF visible around cord and spinal cord compression with no CSF around cord) (Bilsky et al., 2010).

### 2.3 Statistical analysis

Descriptive statistics were performed to summarize patients’ demographic, clinical, radiological, neuropathological, and treatment-related features (Table 1, Table 2, Table 3). All statistical analyses were computed using R (versions 4.2.1 and 4.4.2, R Foundation). Tabular data of the patient cohort, including both categorical and numerical variables, were summarized using the gtsummary package (R Foundation). Median OS (mOS) data was estimated via Kaplan-Meier analysis with 95% CI (Fig. 2A–K, Supplementary Fig. 5A–G), calculated from LMD diagnosis to death or last follow-up. Kaplan-Meier curves were plotted with survminer, with a p-value <0.05 being considered significant (two-sided). Independent prognostic factors for OS were identified using a multivariable Cox regression model which included clinically relevant variables selected for their potential association with survival. The Cox model was generated using stepwise regression, and was computed using the survival and MASS packages. The ggforest function from the survminer package was utilized to visualize the results of the final model (Fig. 3). The waterfall plot (Supplementary Figure 2), illustrating the time from primary diagnosis to LMD diagnosis and from LMD diagnosis to last follow-up as well as the violin plots depicting the time intervals between various diagnosis dates and dates of imaging studies, were generated with ggplot2 (version 3.5.1). The alluvial plot showing the frequency and outcomes of lumbar punctures (LP) across multiple attempts was created using the ggsankey package (version 0.0.9). Additional data handling and visualization steps were carried out using the dplyr package (version 1.1.4), tidyverse meta-package (version 2.0.0), and the viridis package (version 0.6.5) for perceptually uniform color scales.

**Fig. 2:**
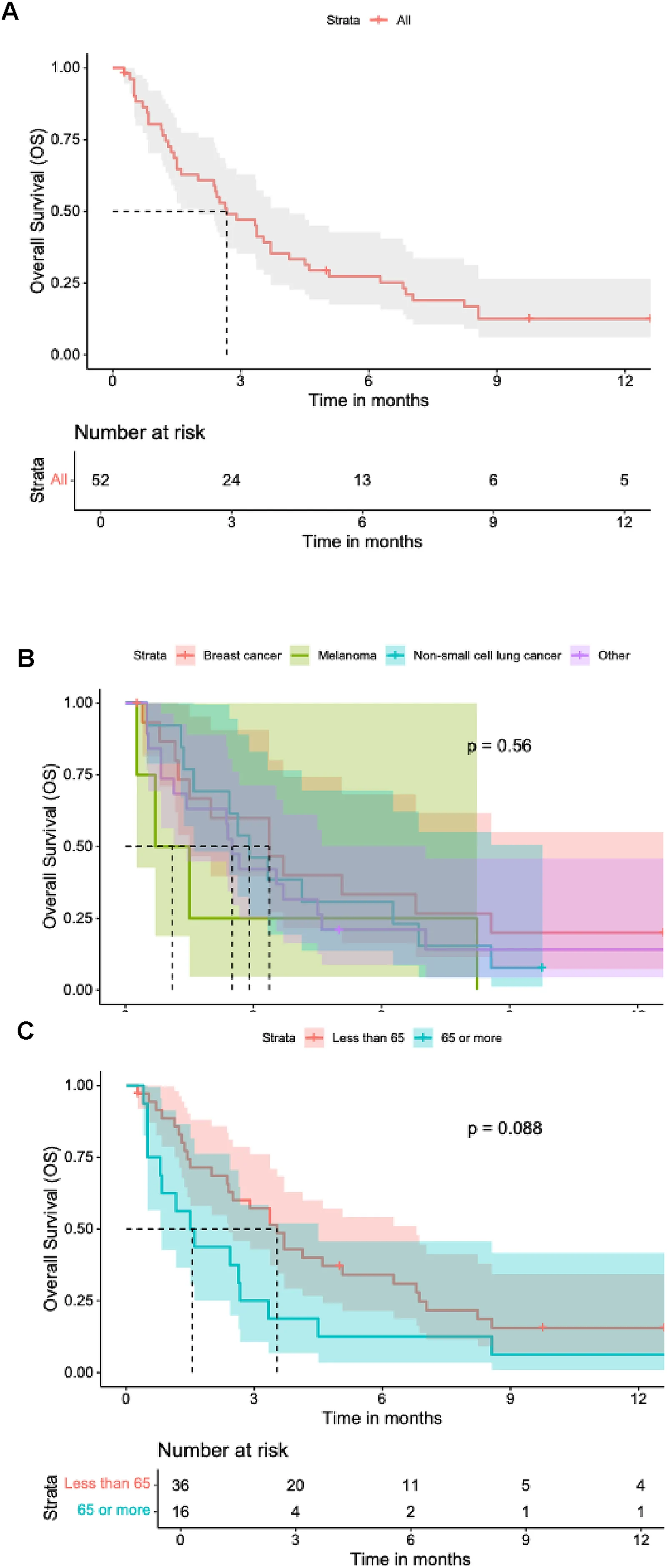

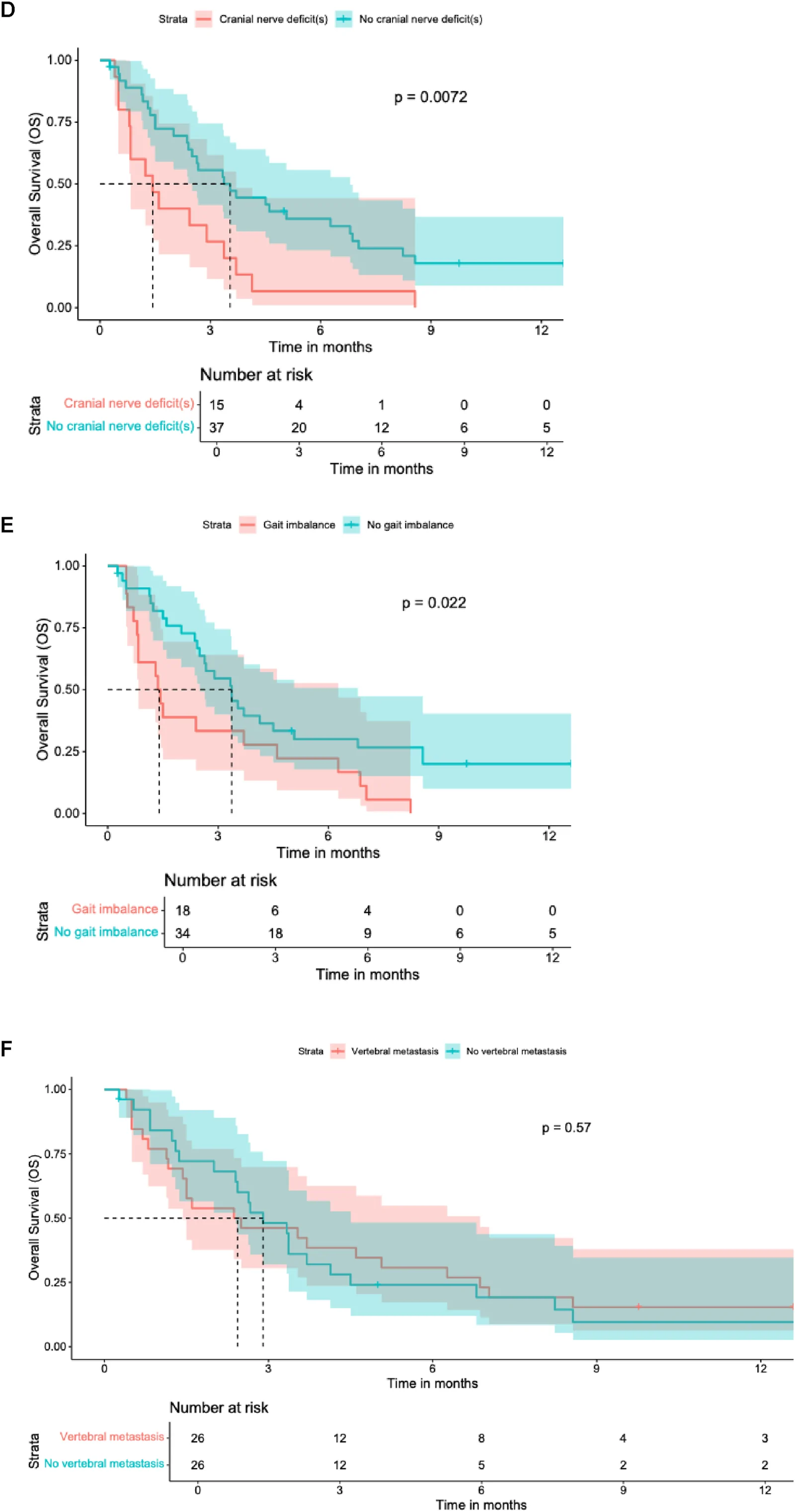

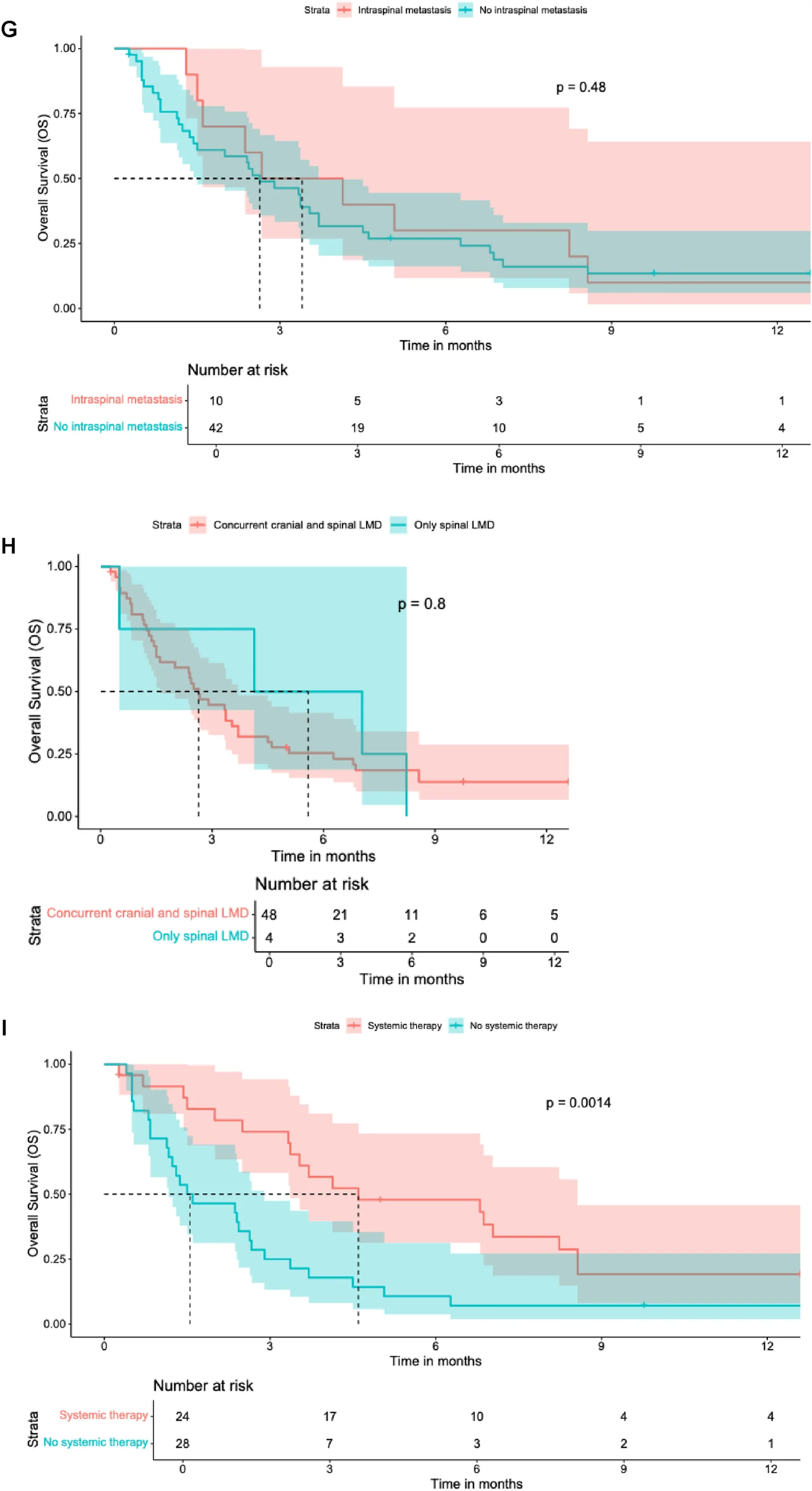

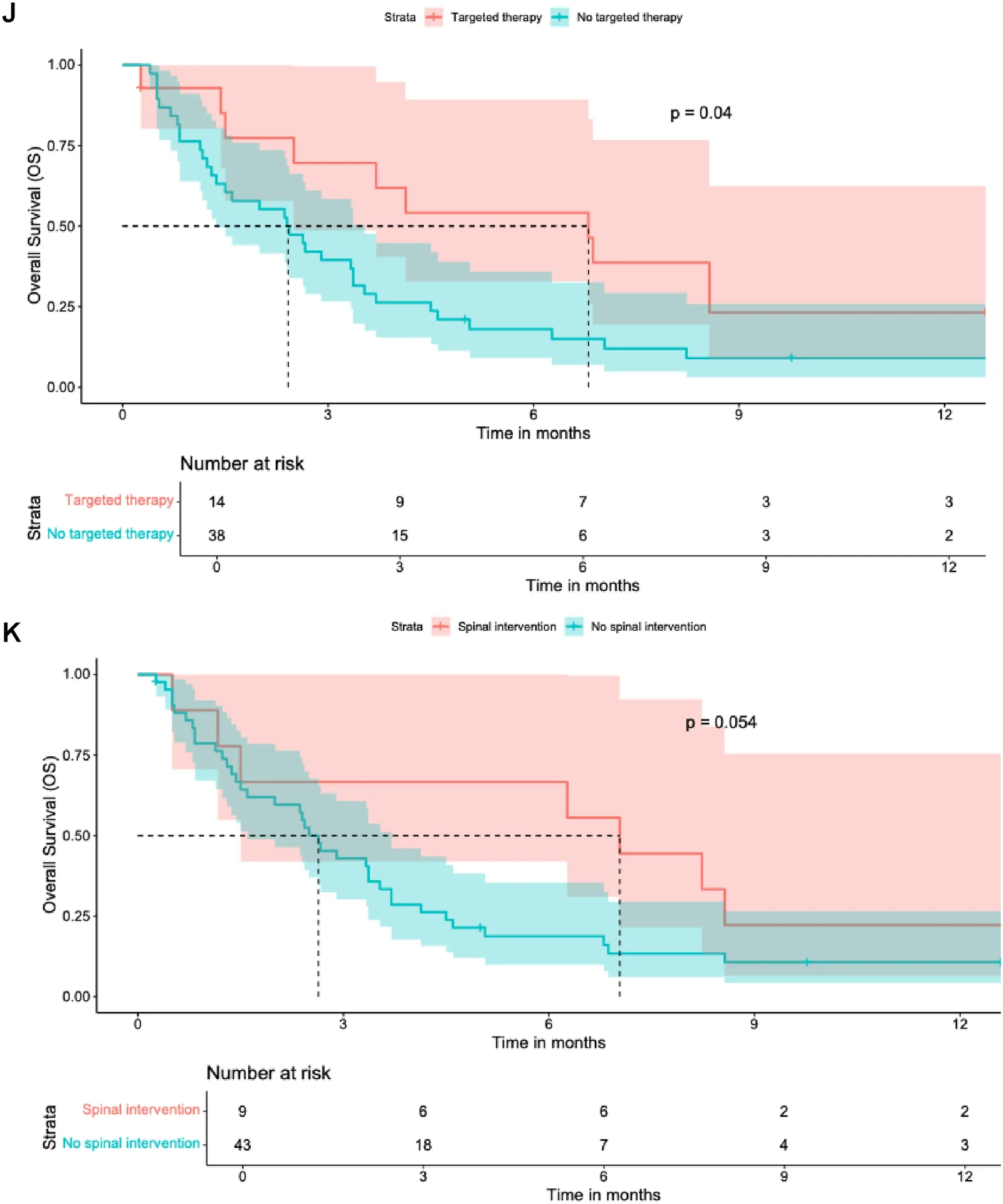
Kaplan-Meier curves estimates for overall survival (OS). Kaplan-Meier curve for the entire cohort (n = 52), showing a median OS (mOS) of 2.7 months [95% CI: 2.0-4.1] (**A**). Kaplan-Meier curves stratified by the three most common primary entities – breast cancer, NSCLC and melanoma – and the remaining 13 entities grouped together. Amongpatients with the three most common primary tumors, breast cancer patients had the longest mOS (3.4 months [1.5-NA]), followed by NSCLC patients (2.9 months [1.6-NA]) and melanoma patients with a notably shorter mOS of 1.1 month [0.3-NA]. Patients with the other 13 primary tumor entities had a mOS of 2.5 months [1.4-7.0]. These differences, however, weren't significant (p = 0.56) (**B**). Kaplan-Meier curves stratified by patients' age (i.e., <65 and ≥ 65) showing that patients ≥65 years old have a mOS of 1.6 months [0.8-4.5] in comparison to patients <65 years old who have a mOS of 3.5 months [2.4-6.8] (p = 0.088) (**C**). Kaplan-Meier curves of the sub-cohorts stratified based on the presence of cranial nerve deficits at LMD diagnosis. Patients without a cranial nerve deficit had a mOS of 3.5 months [2.5-6.8], whereas patients with cranial nerve deficits had a mOS of 1.4 months [0.8-3.7] (p = 0.0072) (**D**). Kaplan-Meier curves of the sub-cohorts stratified based on the presence of gait imbalance at LMD diagnosis. Patients without gait imbalance had a mOS of 3.4 months [2.5-6.8], whereas patients with gait imbalance had a mOS of 1.4 months [0.8-6.3] (p = 0.022) (**E**). Kaplan-Meier curves of the sub-cohorts stratified based on the presence of vertebral metastases at LMD diagnosis. Patients with vertebral metastases had a mOS of 2.4 months [1.4-6.9], whereas those without vertebral metastases had a mOS of 2.9 months [2.4-4.5] (p = 0.57) (**F**). Kaplan-Meier curves of the two sub-cohorts stratified by the presence of intraspinal metastases at LMD diagnosis; the sub-cohort of patients who had at least one intraspinal metastasis at LMD diagnosis had a mOS of 3.4 months [1.6-NA], whereas the median OS of the patients who did not have intraspinal metastases was 2.6 months [1.5-3.7] (p = 0.48) (**G**). Kaplan-Meier curves of the sub-cohorts stratified based the extent of LMD manifestation, i.e., concurrent cranial and spinal LMD or isolated sLMD. Patients with concurrent cranial and spinal LMD had a mOS of 2.6 months [1.6-3.7], whereas those with isolated sLMD had a mOS of 5.6 months [0.5-NA] (p = 0.80) (**H**). Kaplan-Meier curves of the two sub-cohorts stratified by the receipt of systemic therapy after LMD diagnosis; the sub-cohort of patients who have not received any systemic therapy following LMD diagnosis had a mOS of 1.6 months [1.2-2.9], whereas the median OS of the patients who have received systemic therapy following LMD diagnosis was 4.6 months [3.4-8.6] (p = 0.0014) (**I**). Kaplan-Meier curves of the sub-cohorts stratified based on the treatment with TTx after LMD diagnosis. Patients who received TTx following LMD had a mOS of 6.8 months [2.5-NA], whereas those who did not receive TTx had a mOS of 2.4 months [1.4-3.5] (p = 0.04) (**J**). Kaplan-Meier curves of the sub-cohorts stratified based on the performance of spine surgery either before or after LMD diagnosis; patients who have undergone a spinal intervention had a mOS of 7.0 months [1.5-NA], whereas those who did not had a mOS of 2.6 months [1.6-3.7]. This pronounced difference is, with p = 0.054, almost statistically significant (**K**).

**Fig. 3:**
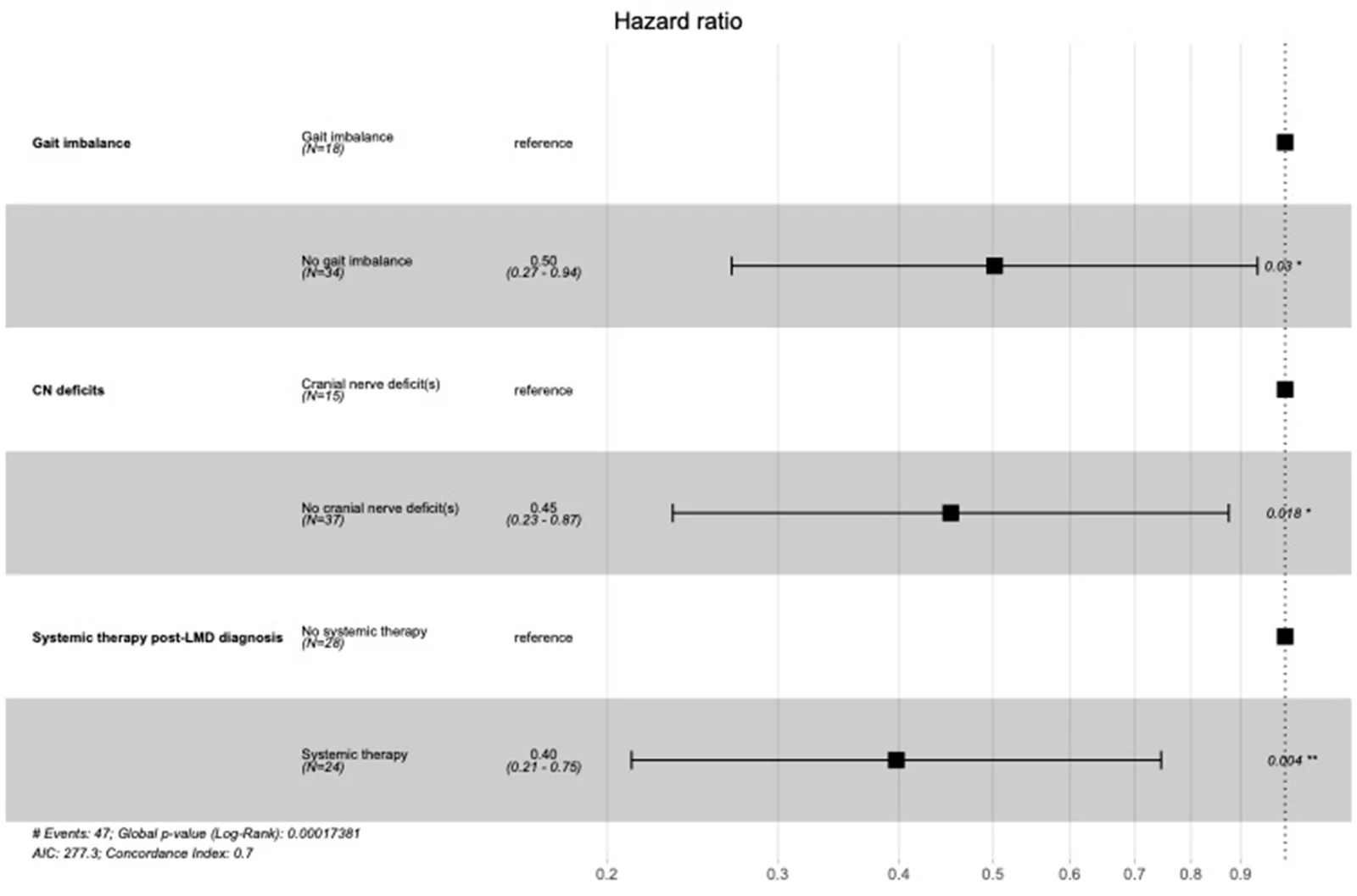
Stepwise Cox regression analysis for overall survival (OS). The forest plot illustrates the hazard ratios (HR) and associated 95% confidence intervals of selected clinical variables included in the final Cox proportional hazards model, which was developed using a backward stepwise selection process. Our model included the primary tumor entity (entity2) and the four factors, which were significant in the univariate analysis – cranial nerve deficits at LMD diagnosis (CN_deficits), gait imbalance at LMD diagnosis (gait_imbalance), systemic therapy following LMD diagnosis (sys_therapy_posLMD_01) and TTx following LMD diagnosis (targeted_therapy_after_dx_of_LMD2). The variables that remained significant in the multivariate model include systemic therapy following LMD diagnosis (HR = 0.40 [95% CI: 0.21-0.75], p < 0.01), absence of cranial nerve deficits (HR = 0.45 [0.23-0.87], p < 0.05) and absence of gait imbalance (HR = 0.50 [0.27-0.94], p < 0.05) at LMD diagnosis. The forest plot confirms the robustness of the stepwise model, where non-significant variables were iteratively removed based on the Akaike Information Criterion (AIC), which balances the goodness of fit and model complexity, ensuring the most parsimonious model without overfitting, thereby providing reliable hazard ratio estimates for the variables that remain. The high concordance index (C = 0.7, SE = 0.043) indicates good predictive accuracy of the model, demonstrating that the selected variables provide a strong explanatory power for the survival data in this patient cohort.

## 3 Results

### 3.1 Baseline patient characteristics

Fifty-two patients with sLMD who underwent cranial and spinal MR imaging within 30 days of diagnosis were included (Fig. 1). The median age at LMD diagnosis was 57.9 years (IQR: 48.8-67.7) and 63.5% of patients were female (Table 1). Median time from primary disease diagnosis to LMD was 30.2 months [95% CI: 18.9-42.6], and from primary diagnosis to death/last follow-up, 32.7 months [24.9-44.3] (Table 1, Supplementary Fig. 2). sLMD incidence increased significantly between 2011 and 2024, with a 15.9% annual rise (coefficient: 0.16, SE = 0.043, z = 3.73, p < 0.001) (Supplementary Figure 3). The most common primary tumors were breast cancer (30.8%), NSCLC (25.0%), and malignant melanoma (7.7%) (Table 1). In total, 16 tumor entities were identified, including rare entities such as alveolar rhabdomyosarcoma and cancer of the ampulla of Vater (Table 1). Targetable alterations were present in 30.8% of tumors, with 84.6% of primary tumors being inactive at LMD diagnosis. The most common symptoms at LMD diagnosis included clinical decline (defined as any decrease in functional status, i.e., any deterioration in physical or cognitive function; 80.8%), headaches (59.6%), gait impairment and nausea (each 34.6%). Spine-specific symptoms included radiculopathy (26.9%) and paraplegia (23.1%). Patients were considered to have a history of parenchymal BrM if their BrM was diagnosed ≥28 days prior to LMD diagnosis. One patient developed a parenchymal BrM following LMD diagnosis. The median time from diagnosis of BrM to LMD diagnosis was 3.3 months [0.7- 4.8]. In 8 patients the BrM was diagnosed over a year prior to LMD diagnosis, whereas the BrM was diagnosed simultaneously to the LMD in 9 patients (Supplementary Figure 4B). Additionally, 36 patients (69.2%) had at least one EcM at LMD diagnosis, and 41 patients (78.8%) had at least one EcM throughout their disease course before LMD diagnosis (Table 1).

**Table 1:** Patient characteristics.

Characteristic	N = 52
**Age, median (IQR)**	57.9 (48.8 – 67.7)
**Sex, n (%)**	
Female	33 (63.5)
Male	19 (36.5)
**Tumor entity, n (%)**	
Breast cancer	16 (30.8)
Melanoma	4 (7.7)
NSCLC	13 (25.0)
Sarcoma	
Alveolar rhabdomyosarcoma	1 (1.9)
Leiomyosarcoma	1 (1.9)
Other	
Biliary cancer	1 (1.9)
Cancer of the ampulla of Vater	1 (1.9)
Cervical cancer	1 (1.9)
Colorectal cancer	1 (1.9)
Esophageal cancer	3 (5.8)
Gastric cancer	1 (1.9)
Ovarian cancer	2 (3.8)
Pancreatic cancer	2 (3.8)
Prostate cancer	1 (1.9)
Renal cell carcinoma	2 (3.8)
Urothelial cancer	1 (1.9)
Cancer of Unknown Primary	1 (1.9)
**Targetable alteration, n (%)**	
Present	
ALK alteration	1 (1.9)
BRAF mutation	2 (3.8)
BRCA1 mutation	1 (1.9)
EGFR mutation	1 (1.9)
HER-2/neu mutation	9 (17.3)
MET alteration	2 (3.8)
Not present	36 (69.2)
**Symptoms at LMD diagnosis, n (%)**	
Headache	31 (59.6)
Nausea	18 (34.6)
Vomiting	10 (19.2)
Vertigo	14 (26.9)
Decline in clinical performance	42 (80.8)
Cognitive deficits	6 (11.5)
Visual deficits	8 (15.4)
Cranial nerve deficits	15 (28.8)
Seizures	2 (3.8)
Gait imbalance	18 (34.6)
Radiculopathy	14 (26.9)
Paraplegia	12 (23.1)
Urinary incontinence	6 (11.5)
**Concurrent cranial LMD and spinal LMD, n (%)**	
Concurrent cranial and spinal LMD	48 (92.3)
Only spinal LMD	4 (7.7)
**Diagnostic modality, n (%)**	
MR imaging	27 (51.9)
MR imaging + CSF cytology	25 (48.1)
**Dominant LMD pattern, n (%)**	
Classical (i.e., MRI subtype A)	24 (46.2)
Nodular (i.e., MRI subtype B)	6 (11.5)
Mixed (i.e., MRI subtype C)	20 (38.5)
No neuroradiological evidence (i.e., MRI subtype D)	2 (3.8)
**EANO ESMO subtype, n (%)**	
IA	14 (26.9)
IB	2 (3.8)
IC	9 (17.3)
IIA	10 (19.2)
IIB	4 (7.7)
IIC	11 (21.2)
IID	2 (3.8)
**KPS at LMD diagnosis, n (%)**	
Bad KPS (<70%)	26 (50.0)
Good KPS (≥70%)	26 (50.0)
**Activity of primary tumor at LMD diagnosis, n (%)**	
Active primary tumor	7 (13.5)
No active primary tumor	44 (84.6)
Not applicable (CUP)	1 (1.9)
**Hydrocephalus at LMD diagnosis, n (%)**	
Hydrocephalus	5 (9.6)
No hydrocephalus	47 (90.4)
**Parenchymal brain metastasis, n (%)**	
Diagnosis before LMD diagnosis	26 (50.0)
Diagnosis at time of LMD diagnosis	9 (17.3)
Diagnosis after LMD diagnosis	1 (1.9)
No parenchymal BrM	16 (30.8)
**Active parenchymal brain metastasis at LMD diagnosis, n (%)**	
Stable disease	13 (25.0)
Progressive disease	22 (42.3)
Not applicable	17 (32.7)
**Brain metastasis resections, n (%)**	
No BrM resection	34 (65.4)
Before LMD diagnosis	13 (25.0)
After LMD diagnosis	4 (7.7)
Before and after LMD diagnosis	1 (1.9)
**Number of brain metastasis resections, n (%)**	
No BrM resections	34 (65.4)
One BrM resection	13 (25.0)
Two BrM resections	5 (9.6)
**History of extracranial metastasis, n (%)**	
History of EcM	41 (78.8)
No history of EcM	11 (21.2)
**Extracranial metastasis at LMD diagnosis, n (%)**	
Presence of at least one EcM	36 (69.2)
No EcM	16 (30.8)
**Activity of extracranial metastasis at LMD diagnosis, n (%)**	
Partial response	4 (7.7)
Stable disease	19 (36.5)
Progressive disease	20 (38.5)
Unknown	9 (17.3)
**Cause of death, n (%)**	
CNS progression	17 (32.7)
Extra-CNS progression	1 (1.9)
Other	2 (3.8)
Unknown	27 (51.9)
Not applicable (i.e. alive)	5 (9.6)

Patient characteristics including demographic and clinical characteristics as well as neuropathological and imaging-related features. Based on radiological findings, LMD was described as either classical or nodular according to the classification by Turner et al. and further classified into MRI subtypes following the EANO ESMO recommendation (Le Rhun et al., 2017b; Turner et al., 2020).

### 3.2 Diagnosis of cranial and spinal disease

LMD was diagnosed in 51.9% of cases using MR imaging exclusively and in 48.1% of cases through both MRI and CSF cytology analysis (Table 1). Seventeen patients (32.7%) got their cranial and spinal imaging on the same day, 24 patients (46.2%) had an interval of 1 to 10 days, 7 patients (13.5%) an interval of between 10 and 20 days, and the remaining 4 patients (7.7%) had an interval of between 20 and 30 days between their cranial and spinal MRI (sMRI) (Supplementary Figure 4C). Only 12 patients (23.1%) received follow-up spinal imaging after the initial sMRI with which sLMD was confirmed. The median number of follow-up images in those patients was 1.5 (IQR: 1-2.5) (Table 2), with a moderate positive correlation between the number of follow-up sMRIs and patients' OS (Kendall's τ = 0.38, p = 0.0006). Based on the imaging, 48 patients (92.3%) had concurrent cranial and spinal LMD manifestation at diagnosis, whereas 4 patients (7.7%) had isolated spinal manifestation (Table 1). Almost a half of all patients (46.2%) had a linear LMD pattern (i.e., MRI subtype A), 20 patients (38.5%) had a linear and nodular pattern (i.e., MRI type C) and 6 patients (11.5%) had a nodular LMD pattern (i.e., MRI type B) (Table 1, Supplementary Figure 1). In total, 37 (71.2%) of the patients in our cohort have undergone at least one LP in the context of LMD diagnosis. Data on serial LPs show that on the first LP (LP1), 22 out of 37 patients (59.5%) tested positive, whereas on the second LP (LP2), 7 out of 16 patients (43.8%) were positive, and by the third LP (LP3), the percentage of samples in which tumor cells were found decreased to 28.6% (2 out of 7 patients). Of the patients who received at least one LP, tumor cells could be identified in only 67.6%. The 12 patients in whose CSF tumor cells could not be identified underwent a median of 2 LPs (IQR: 1-3)(Supplementary Figure 4D).

**Table 2:** Spine-specific patient features.

Characteristic	N = 52
**Follow-up spinal imaging, n (%)**	
No	40 (76.9)
One sMRI	6 (11.5)
Two sMRIs	3 (5.8)
Three sMRIs	1 (1.9)
Four sMRIs	2 (3.8)
**Timepoint of vertebral metastasis diagnosis, n (%)**	
Before LMD diagnosis	17 (32.7)
At LMD diagnosis	9 (17.3)
After LMD diagnosis	4 (7.7)
No vertebral metastases	22 (42.3)
**Vertebral metastases at LMD diagnosis, n (%)**	
ESCC 0	19 (36.5)
ESCC 1a	1 (1.9)
ESCC 3	1 (1.9)
Unknown	5 (9.6)
No vertebral metastases	26 (50.0)
**Presence of intraspinal metastases at LMD diagnosis, n (%)**	
Yes	10 (19.2)
No	42 (80.8)
**Radiation therapy of the spine, n (%)**	
Before LMD diagnosis	8 (15.4)
After LMD diagnosis	13 (25.0)*
Before and after LMD diagnosis	3 (5.8)
No RTx of the spine	26 (50.0)
Unknown	2 (3.8)
**Spine surgeries, n (%)**	
Before LMD diagnosis	
Dorsal instrumentation following a vertebral fracture	1 (1.9)
Extended spinal decompression + dorsal instrumentation	1 (1.9)
Kyphoplasty for an osteolytic metastasis and a vertebral compression	1 (1.9)
fracture	
Biopsy of intraspinal metastasis and dorsal instrumentation	1 (1.9)
corpectomy and metastasis resection with bilateral facetectomy,	
metastasis re-resection (following embolization), spinal canal	
decompression and pedicle replacement, revision following rod	
breakage	
Following LMD diagnosis	
Hemilaminectomy	2 (3.8)
Laminectomy	1 (1.9)
Extended interlaminar fenestration	1 (1.9)
Posterior fixation following a vertebral fracture	1 (1.9)
No spine surgeries	43 (82.7)

Spine-specific features including follow-up sMRI, presence of vertebral and/or intraspinal metastases before and at LMD diagnosis, the extent to which vertebral metastases compromise the spinal canal at LMD diagnosis (assessed using the ESCC scale (Kokkoris, 1983)) and spinal surgeries. The ESCC grade of 5 patients (14.7%) could not be determined due to lack of axial imaging at LMD diagnosis. *Two of the patients in our cohort who underwent spinal RTx following LMD diagnosis discontinued their therapy due to side effects, as these patients did receive at least a part of their RTx they were included in the “RTx of the spine” group.

### 3.3 Spinal metastases and spinal leptomeningeal disease

Twenty-four patients (46.2%) had vertebral metastases, 4 (7.8%) had intraspinal metastases, and 6 (11.5%) had both (Table 2). Among those with vertebral metastases, 56.7% were diagnosed before, 30% at, and 13.3% after LMD diagnosis (Table 2). The median time from spinal/intraspinal metastasis diagnosis to LMD was 0.1 months [0.0-5.4]. In 37.5%, metastases were diagnosed within a year before LMD, in 9.4% between 1 and 2 years, in 6.3% between 3 and 4 years, and in another 6.3% over 5 years before. Most patients (73.1%) diagnosed with vertebral metastases before or at LMD diagnosis had ESCC grade 0 spine metastases at LMD diagnosis, while one patient (3.8%) had grade 1a and 3, respectively. In 5 patients (19.2%), ESCC grading was undetermined due to missing axial imaging (Table 2). Notably, 50.0% of the entire cohort did not receive spinal RTx. Among those who did, 33.3% received it before, 54.2% after, and 12.5% both before and after LMD diagnosis (Table 2). Spine surgery was performed in 26.5% of patients with vertebral/intraspinal metastases; 44.4% had surgery before LMD, and 55.6% after. Pre-diagnosis surgeries included a biopsy of an intraspinal metastasis, metastasis resections, spinal decompressions, dorsal instrumentations, and a kyphoplasty, with 3 patients undergoing a single procedure and one patient undergoing 6 different procedures. Post-diagnosis surgeries (all single procedures) included hemilaminectomy (40%), laminectomy (20%), and extended interlaminar fenestration (20%) and posterior fixation following a vertebral fracture (20%)(Table 2).

### 3.4 Local and systemic therapies before LMD diagnosis

Prior to LMD diagnosis, 31 patients (59.6%) received local therapy, and 43 patients (82.7%) received systemic therapy (Table 3). At the time of LMD diagnosis, 35 patients (67.3%) had active parenchymal BrMs, and 26 patients (50.0%) had a history of parenchymal BrM, of which 14 patients (26.9% of entire cohort, 53.8% of patients with history of BrMs) have undergone at least one microsurgical resection previously (Table 1). Among the patients who received local therapy, 45.2% underwent a BrM resection, 12.9% underwent a spinal intervention, 67.7% received cranial RTx, and 35.5% received spinal RTx. Seventeen patients received multiple local therapy modalities; the combinations included a microsurgical BrM resection and RTx of the cranial compartment (70.6% of those receiving multiple local therapy modalities), cranial and spinal RTx (11.8%), a spinal intervention and spinal RTx (11.8%), and maximal therapy including a BrM resection, spinal intervention as well as cranial and spinal RTx (5.9%) (Table 3). Of the 14 patients who have undergone a BrM resection prior to LMD diagnosis, 11 have undergone a single resection and 3 patients have undergone 2 resections (Table 3). Of the patients who have received systemic therapy, most (51.2%) were on their first line of therapy at LMD diagnosis, 11.6% were on their second, 20.9% were on their third, and 16.3% received four or more lines of therapy. The most common systemic therapy regimens included chemotherapy (CTx) alone (30.2%), followed by a combination of CTx and targeted therapy (TTx) (25.6%) and a combination of CTx, TTx and hormone therapy (16.3%) (Table 3).

**Table 3:** Local, systemic and associated treatments before and after LMD diagnosis.

Characteristic (N = 52)	Before LMD Diagnosis	After LMD Diagnosis
**Surgical therapy, n (%)**		
BrM resection	13 (25.0)	4 (7.7)
Spinal intervention	3 (5.8)	4 (7.7)
BrM resection + spinal intervention	1 (1.9)	1 (1.9)
No surgical therapy	35 (67.3)	43 (82.7)
**Number of brain metastasis resections, n (%)**		
No resection	39 (75.0)	48 (92.3)
One	10 (19.2)	3 (5.8)
Two	3 (5.8)	1 (1.9)
**Radiation therapy, n (%)**		
Cranial RTx	18 (34.6)	8 (15.4)
Spinal RTx	8 (15.4)	3 (5.8)
Cranial + spinal RTx	3 (5.8)	11 (21.2)
Craniospinal RTx	0 (0.0)	2 (3.8)
No RTx	23 (44.2)	28 (53.8)
**Cranial radiation therapy, n (%)**		
Conventional RTx	2 (3.8)	2 (3.8)
Stereotactic radiosurgery (SRS)	9 (17.3)	0 (0.0)
Whole-brain radiation (WBRT)	4 (7.7)	16 (30.8)
WBRT + SRS	4 (7.7)	1 (1.9)
Craniospinal irradiation	0 (0.0)	2 (3.8)
Not available	2 (3.8)	0 (0.0)
No cranial RTx	31 (59.6)	31 (59.6)
**Spinal radiation therapy, n (%)**		
Conventional RTx	9 (17.3)	14 (26.9)
SRS	1 (1.9)	0 (0.0)
Not furthered classified	1 (1.9)	0 (0.0)
Craniospinal irradiation	0 (0.0)	2 (3.8)
Not available	2 (3.8)	2 (3.8)
No spinal RTx	39 (75.0)	34 (65.4)
**Systemic therapy modalities, n (%)**		
Chemotherapy (CTx)	13 (25.0)	6 (11.5)
Hormone therapy	1 (1.9)	1 (1.9)
Immunotherapy (CPI)	4 (7.7)	3 (5.8)
Targeted therapy (TTx)	0 (0.0)	8 (15.4)
CTx + hormone therapy	3 (5.8)	0 (0.0)
CTx + CPI	1 (1.9)	0 (0.0)
CTx + TTx	11 (21.1)	4 (7.7)
CTx + CPI + TTx	1 (1.9)	0 (0.0)
CTx + TTx + hormone therapy	7 (13.5)	1 (1.9)
CTx + CPI + TTx + hormone therapy	1 (1.9)	0 (0.0)
CPI + TTx	1 (1.9)	1 (1.9)
No systemic therapy	9 (17.3)	28 (53.8)
**Ventriculoperitoneal shunt (VPS), n (%)**		
VPS implanted	2 (3.8)	4 (7.7)
No VPS implanted	50 (96.2)	48 (92.3)
**Reservoir, n (%)**		
Reservoir implanted	0 (0.0)	6 (11.5)
No reservoir implanted	52 (100.0)	46 (88.5)
**Methotrexate intrathecal therapy, n (%)**		
Via repetitive LP	0 (0.0)	13 (25.0)
Through a reservoir	0 (0.0)	6 (11.5)
No	52 (100.0)	33 (63.5)

Treatment-related patient characteristics before and after LMD diagnosis including local and systemic treatments as well as interventions performed for the management of an associated hydrocephalus (e.g., implantation of a VPS).

### 3.5 Local and systemic therapies following LMD diagnosis

Following LMD diagnosis, 27 patients (51.9%) received local therapies (Table 3), including cranial RTx in 21 patients (77.8% of patients receiving local therapy) and spinal RTx in 16 patients (59.3%). Of those, 13 patients received both cranial and spinal RTx or RTx of the craniospinal axis. BrM microsurgical resection was performed in 4 patients (7.7% of the entire cohort, 14.8% of patients receiving local therapy) – three underwent a single resection and one underwent two resections. One of these patients had also undergone a BrM resection prior to LMD diagnosis. Moreover, 5 patients underwent spinal surgery (Table 3). Six patients (11.5%) had a reservoir, and 4 patients (7.7%) had a ventriculoperitoneal shunt (VPS) implanted after LMD diagnosis (Table 3). Systemic therapy was administered to 24 patients (46.2%), mostly either TTx (33.3% of cases), CTx (25.0%), a combination of CTx and TTx (16.7%), or checkpoint inhibitors (CPI) (12.5%) (Table 3). Additionally, 19 patients (36.6%) received intrathecal methotrexate, mostly (68.4%) via repetitive LPs (Table 3).

### 3.6 Patient outcomes and association of clinical factors with OS

The median follow-up time was 2.7 months [2.0-3.7], with 47 deaths (90.4%) during the follow-up period. The mOS following LMD diagnosis was 2.7 months [2.0-4.1] and from primary disease diagnosis was 32.7 months [23.7-45.0] (Table 1, Fig. 2A–Supplementary Table 1). Among patients with the three most common primary tumors, breast cancer patients had the longest mOS (3.4 months [1.5-NA]), followed by NSCLC (2.9 months [1.6-NA]) and melanoma patients (1.1 months [0.3-NA]). Patients with the other 13 primary entities had a mOS of 2.5 months [1.4-7.0]. These differences, however, were not statistically significant (p = 0.56) (Fig. 2B,Supplementary Table 1). Patients <65 years had a longer mOS (3.5 [2.4-6.8]) than those ≥65 years (1.6 [0.8-4.5]), though this difference was also statistically insignificant (p = 0.088) (Fig. 2C,Supplementary Table 1). Most symptoms were not associated with OS, yet patients with cranial nerve deficits had a significantly shorter mOS than patients without (1.4 months [0.8-3.7] vs. 3.5 months [2.5-6.8], p = 0.0072) as did those with gait imbalance (1.4 months [0.8-6.3] vs. 3.4 months [2.5-6.8], p = 0.022) (Fig. 2D and E,Supplementary Table 1). Additional common clinical variables investigated in LMD patients, such as KPS at LMD diagnosis, presence of targetable alterations or presence of a hydrocephalus at LMD diagnosis were not associated with OS (Supplementary Fig. 5A-5C; Supplementary Table 1). Among patients with a documented cause of death, CNS progression was the most frequently recorded cause (Table 1).

The activity of primary disease at LMD diagnosis was not associated with mOS (p = 0.93) (Supplementary Fig. 5D, Supplementary Table 1**)** as were neither the presence nor the activity of parenchymal BrM and EcM at the time of LMD diagnosis nor a history of parenchymal BrM or EcM (Supplementary Table 1). Similarly, vertebral metastases (2.4 months [1.4-6.9] vs. 2.9 months [2.4-4.5], p = 0.57) and intraspinal metastases (3.4 months [1.6-NA] vs. 2.6 [1.5-3.7], p = 0.48) had no significant association with OS as well (Fig. 2F and G, Supplementary Table 1).

Patients in whose CSF tumor cells could not be identified (EANO ESMO type II) had a longer mOS (3.4 months [2.5-7.0]) than patients with tumor cells positive CSF (EANO ESMO type I) (1.8 months [1.2-3.7]), though this association was not statistically significant (p = 0.081) (Supplementary Fig. 5E, Supplementary Table 1).The MRI subtype was also not associated with OS (p=0.55). Patients with type A manifestation (linear LMD) had a mOS of 1.6 months [1.2-6.3], whereas those with type B (nodular LMD) had a mOS of 2.8 months [2.5-NA] and patients with type C (mixed LMD) had a mOS of 3.6 months [2.0-8.6](Supplementary Fig. 5F, Supplementary Table 1). No significant difference in OS was found between patients with concurrent cranial and spinal LMD (2.6 months [1.6-3.7]) and those with isolated sLMD (5.6 months [0.5-NA]) (p = 0.80) (Fig. 2H,Supplementary Table 1).

No significant difference in OS was observed between treatment-naïve and pre-treated patients (2.9 months [0.8-NA] vs. 2.7 months [2.0-4.5], p = 0.72) (Supplementary Fig. 5G, Supplementary Table 1), nor was OS associated with the number of prior systemic therapy lines or RTx pre-treatment. Any systemic treatment pre-LMD diagnosis, except CPI, was associated with increased survival, yet none of those associations reached statistical significance (p = 0.15-0.84). Neither cranial nor spinal RTx pre-LMD diagnosis were associated with a survival benefit. Even though all local therapy modalities post-LMD diagnosis were associated with an increased survival, none of them reached statistical significance (p = 0.14-0.51). Systemic therapy post-LMD, however, was associated with a significantly prolonged OS (4.6 months [3.4-8.6] vs. 1.6 months [1.2-2.9], p = 0.0014) (Fig. 2I,Supplementary Table 1), though no single treatment modality, except TTx, conferred a significant survival advantage. Patients receiving TTx post-LMD had a mOS of 6.8 months [2.5-NA] in comparison to 2.4 months [1.4-3.5] in those who did not (p = 0.04) (Fig. 2J,Supplementary Table 1). Patients who underwent at least one spinal intervention either before or after LMD diagnosis (n = 9) had a mOS of 7.0 months [1.5-NA] in contrast to a mOS of 2.6 months [1.6-3.7] among those who did not; this pronounced difference is, with p = 0.054, almost statistically significant (Fig. 2K,Supplementary Table 1).

### 3.7 Independent prognostic factors

In order to identify independent prognostic factors for OS, we performed a backward stepwise multivariate Cox proportional hazards regression for various parameters, including the entity of the underlying primary tumor and the factors which showed statistical significance in the univariate analysis – cranial nerve deficits at LMD diagnosis, gait imbalance at LMD diagnosis, systemic therapy following LMD diagnosis and TTx following LMD diagnosis. Independent prognostic factors for improved survival included systemic therapy following LMD diagnosis (HR = 0.40 [0.21-0.75], p < 0.01), absence of cranial nerve deficits (HR = 0.45 [0.23-0.87], p < 0.05) at LMD diagnosis and absence of gait imbalance (HR = 0.50 [0.27-0.94], p < 0.05) at LMD diagnosis (Fig. 3). The model demonstrates a high concordance index (C = 0.7, SE = 0.043), indicating good predictive accuracy and that the selected variables provide a strong explanatory power for the survival data in this patient cohort.

## 4 Discussion

This single-center cohort study offers a comprehensive characterization of solid tumor patients with newly diagnosed sLMD, who constitute a particularly understudied subset of patients with LMD. Our findings provide detailed insights into the clinical presentation, diagnosis, course of disease, treatment, and outcomes of sLMD patients, underscoring the diagnostic limitations and clinical challenges in managing this disease manifestation.

Most patients’ primary disease was inactive at the time of LMD diagnosis and less than half of all patients had BrM or EcM progression, respectively, suggesting that the dissemination of tumor cells to the meninges is likely to occur through different routes (e.g., hematogenous dissemination via systemic circulation, spread from adjacent metastatic lesions) in different patients (Freret and Boire, 2024; Remsik and Boire, 2024; Yue et al., 2025; Ozair et al., 2024; Sener et al., 2025). Neither the activity of primary disease at LMD diagnosis, nor history of parenchymal BrM or EcM before LMD diagnosis, nor the presence of parenchymal BrM and EcM or the activity of parenchymal BrM and EcM at LMD diagnosis were associated with decreased OS, indicating that LMD is the predominant factor contributing to patient mortality even in patients with extensive systemic and solid CNS disease. Moreover, neither the presence of vertebral metastases at LMD diagnosis nor the timing of vertebral metastases diagnosis in relation to LMD diagnosis had a significant association with OS. Given the small number of patients with infiltrating vertebral metastases (i.e., vertebral metastases with an ESCC grade >0), we could not explore the significance of ESCC grade (i.e., extent of spinal canal invasion) on OS, which could shed light on the importance of dissemination of cancer cells originating in vertebral metastases into the spinal canal. The presence of intraspinal metastasis at LMD diagnosis, however, was not associated with poorer survival outcomes.

In our cohort, LMD diagnosis relied primarily on imaging, with CSF cytology confirming the diagnosis in only 48.1% of cases. This highlights limitations in current diagnostic practices, including the lack of regular serial CSF sampling, the low positivity rate of a single LP in detecting tumor cells, and the need for improved diagnostic workflows, potentially involving advanced imaging techniques or liquid biopsies to enhance diagnostic sensitivity (Ferguson et al., 2021; Sener et al., 2021; Dho et al., 2026). Given the retrospective nature of the study, the lack of routine documentation of CSF volume collected at each LP, and potential examiner-, patient- and procedure-related variability in the volume obtained, the exact volume of CSF analyzed in each cytological examination could not be determined. It, therefore, cannot be excluded that the relatively low sensitivity observed in our cohort was, at least in part, attributable to insufficient CSF volume in some examinations. Similarly to the finding of Le Rhun et al. regarding the prognostic value of the EANO ESMO classification of LMD, patients with EANO ESMO type I LMD (i.e., positive CSF cytology) had worse OS than patients with type II (i.e., clinical and/or neuroradiological evidence with negative CSF cytology), even though this association was insignificant in our cohort. Due to the small size of each MRI subtype sub-cohort, we could not investigate its prognostic value in patients with different EANO ESMO subtypes (Le Rhun et al., 2021). The detection of tumor cells in LP1 (i.e., LP1 positivity), which is likely associated with a higher circulating tumor cell (CTC) burden and extent of LMD, was associated with a shorter OS, although this association did not reach statistical significance (p = 0.11). This finding is inconsistent with the results of van Kessel et al. (van Kessel et al., 2025), who showed that the CTC count in CSF of patients with LMD from epithelial tumors is of significant prognostic value. This discrepancy may be a result of our relatively small sample size. It is nonetheless also important to note, that while van Kessel et al. analyzed CTC number, we evaluated LP1 positivity as a binary parameter, and hence the two results are not directly comparable.

Additionally, adherence to the 2017 EANO ESMO Clinical Practice Guidelines for diagnosis, treatment and follow-up of patients with leptomeningeal metastasis from solid tumors was suboptimal (Le Rhun et al., 2017b, 2019). For example, only 23.1% of our cohort received follow-up spinal imaging, raising concerns about the adequacy of disease monitoring and consideration of radiological disease progression and therapy response in treatment decision-making. While the short OS following LMD diagnosis likely contributes to this low follow-up rate, current clinical guidelines (e.g., EANO ESMO Clinical Practice Guidelines) recommend routine follow-up examinations despite the disease's poor prognosis (Le Rhun et al., 2017b). The recommended follow-up encompasses a detailed neurological examination and cerebrospinal MR imaging every 2 months for the first 6 months and every 3 months thereafter in patients with stable disease. Moreover, clinical neurological examinations should also be carried out at emergence of new neurological symptoms or evidence of radiological progression, and an MRI of the neuroaxis should be performed upon suspicion of clinical progression (Le Rhun et al., 2017b, 2019). Additionally, patients undergoing intrathecal therapy should undergo CSF cytology studies every two tothree months as well (Le Rhun et al., 2017b). These recommendations, however, are based on consensus and expert opinions rather than robust data, highlighting the need for studies examining the utility of these different modalities and different follow-up protocols. We believe that the follow-up strategy should be individualized according to the patients' clinical status, treatment approach, and the potential consequences of disease progression. For patients who are considered candidates for further, potentially disease-modifying therapy, we would favor relatively short-interval clinical and imaging follow-up. Given LMD's rapid progression and the limited survival in the absence of effective disease control, we believe that repeat cerebrospinal MR imaging 4-8 weeks after initiation of therapy would be reasonable, particularly when imaging findings are to inform treatment decisions. Subsequent imaging intervals should be adapted to the individual clinical course, treatment response, and therapeutic options, and could be further stratified based on prognostic factors.

The management of sLMD in our cohort exhibited significant variability, reflecting the lack of standardized treatment protocols for this challenging condition. Before LMD diagnosis, 59.6% of patients received local therapies and 82.7% systemic therapies, while post-diagnosis, local therapies were administered in 51.9% and systemic therapies in 46.2% of cases. Despite the availability of intrathecal therapies such as methotrexate, only 36.5% of patients received such treatment, highlighting the ongoing uncertainty regarding optimal therapeutic approaches.

Notably, 92.3% of patients presented with concurrent cranial and spinal LMD, underscoring the systemic nature of the disease and the necessity for a comprehensive treatment strategy. Interestingly, we observed no significant difference in OS between patients <65 years and those ≥65 years as well as patients with good (KPS ≥70%) and poor (KPS <70%) performance status, suggesting that in our cohort these factors may have had limited prognostic value. This may be explained by the rapidly progressive nature of LMD, which may substantially determine the clinical course irrespective of patients’ age or functional status at LMD diagnosis. The rapid disease progression may likewise explain the lack of statistically significant associations between the activity of primary disease at LMD diagnosis as well as history, presence and activity of parenchymal BrM or EcM at LMD diagnosis, and the presence and timing of vertebral metastasis, and overall survival in our cohort as LMD leads to neurological deterioration, the loss of vital functions and eventually death faster than the aforementioned factors, limiting their prognostic value. Nonetheless, these findings should be interpreted with caution, particularly in the context of their divergence from other findings in current literature, as the absence of statistically significant associations between these factors and OS may also reflect the relatively small sample size of our cohort. However, patients who received any form of post-diagnosis treatment – whether local or systemic – demonstrated improved OS, suggesting that active management may confer survival benefits. Among the treatment modalities, systemic therapy and targeted therapy, in particular, were significantly associated with prolonged OS. Given the limited follow-up imaging and the overall poor prognosis of sLMD, further multicenter studies are necessary to refine prognostic models, establish standardized treatment pathways, and determine the precise role of surgical interventions in disease management. A prospective evaluation of treatment response and functional outcomes will be essential to optimize clinical decision-making and improve patient care.

The median survival of 2.7 months in our cohort aligns with existing data on LMD, emphasizing the poor prognosis despite advances in cancer care (Yue et al., 2025; Ozair et al., 2024; Sener et al., 2025; Le Rhun and Galanis, 2016; Le Rhun et al., 2017a; Le Rhun et al., 2017b; Taillibert and Chamberlain, 2018; Bonig et al., 2019; Ferguson et al., 2021; Wilcox et al., 2024; Thakkar et al., 2020; Nayar et al., 2017; Roy-O'Reilly et al., 2023; Pellerino et al., 2021; Le Rhun et al., 2019; Palmisciano et al., 2022; Nguyen et al., 2021; Dankner et al., 2021; Nguyen et al., 2020; Mack et al., 2016; Wang et al., 2018; Wang et al., 2025; Smith et al., 2024). The predominance of breast cancer, NSCLC, and melanoma aligns with epidemiological trends of LMD described in the literature (Yue et al., 2025; Sener et al., 2025; Le Rhun and Galanis, 2016; Le Rhun et al., 2017a; Le Rhun et al., 2017b; Taillibert and Chamberlain, 2018; Ferguson et al., 2021; Wilcox et al., 2024; Thakkar et al., 2020; Nayar et al., 2017; Roy-O'Reilly et al., 2023; Pellerino et al., 2021; Le Rhun et al., 2019; Palmisciano et al., 2022). However, our focus on sLMD highlights distinct disease patterns and management challenges, differentiating it from cranial LMD and emphasizing the need for targeted research. Clinicians should adhere to the EANO ESMO guidelines, incorporating cerebrospinal imaging upon clinical suspicion of LMD and during follow-up of cranial LMD to detect spinal involvement (Le Rhun et al., 2017b).

This study's retrospective, single-center design and the heterogeneity of primary malignancies limit its generalizability. The small sample size and short median follow-up may have constrained the detection of some associations. An additional limitation is the relatively strict inclusion criteria, which may have led to the exclusion of atypical or underdiagnosed cases leading to selection bias. Moreover, patients who survive longer are more likely to receive multiple treatments, especially following LMD diagnosis, potentially leading to survivorship bias and immortal time bias, which may have, in turn, lead to the overestimation of the benefit of some post-diagnosis treatments. The low follow-up rates (e.g., 23.1% had spinal imaging) hindered a comprehensive characterization of systemic, cranial, and spinal disease progression post-LMD diagnosis.

## 5 Conclusions

Our findings underscore the clinical challenges, diverse treatments, and poor prognosis of sLMD. By highlighting the systemic nature of LMD, its primary contribution to patient mortality, shortcomings in current diagnostic workflows, lack of standardized efficacious treatment, and dismal survival outcomes, this study lays a foundation for further research aimed at shaping clinical guidelines, identifying key determinants for therapy decisions, developing new targeted therapies for LMD, and improving management strategies and outcomes for this patient cohort.

## Ethics approval

This retrospective study was approved by the local institutional review board (ethic registration number: EA1/292/23). Due to the study's retrospective nature, which entails no additional risk to the participating patients, informed consent was institutionally waived.

## Authorship

Conceptualization (Z.S., D.W., M.M., P.V.), data curation (Z.S., C.E., C.J., A.F. and D.W.), statical analysis (Z.S. and D.W.), drafting the manuscript (Z.S. and D.W.), providing access to clinical data (J.N., U.K., M.M., P.V. and D.W.), review and editing (Z. S., C. E., C.J., A.F., R.X., H.R., J.N., F.E, G.A., D.Z., L.W., U.K., J.O., M.M., P.V., D.W.).

## Data availability

Anonymized clinical data and R code will be made available upon reasonable request to the authors.

## Funding

This research was not supported by specific grants.

## Declaration of competing interest

The authors declare the following financial interests/personal relationships which may be considered as potential competing interests: Zoe Shaked reports a relationship with European Academy of Neurology that includes: travel reimbursement. Zoe Shaked reports a relationship with German Cancer Society that includes: travel reimbursement. Felix Ehret reports a relationship with German Cancer Aid that includes: funding grants and travel reimbursement. Felix Ehret reports a relationship with Accuray Inc that includes: funding grants, speaking and lecture fees, and travel reimbursement. Felix Ehret reports a relationship with ZAP Surgical Systems, Inc. That includes: travel reimbursement. Felix Ehret reports a relationship with the German Society of Radiation Oncology (jDEGRO Trial Group Chair), Radiosurgery Society (RSS Resident Executive Board Chair), European Organisation for Research and Treatment of Cancer (EORTC Brain Tumor Group Steering Committee Member, Young & Early Career Investigator Radiation Oncology Scientific Council Steering Committee Member), European Association of Neuro-Oncology (Guidelines Committee Member), European Society for Radiotherapy and Oncology (CNS Focus Group Member), and Neuro-Oncological Working Group of the German Cancer Society (Young NOA Steering Committee Member). Daniel Zips reports a relationship with Varian Medical Systems Inc that includes: funding grants and non-financial support. Daniel Zips reports a relationship with Accuray Inc that includes: funding grants and non-financial support. Daniel Zips reports a relationship with Sennewald that includes: funding grants and non-financial support. Ulrich Keilholz reports a relationship with Owkin that includes: funding grants and speaking and lecture fees. Ulrich Keilholz reports a relationship with Cure51 that includes: funding grants. Ulrich Keilholz reports a relationship with Novartis that includes: travel reimbursement. Martin Misch reports a relationship with Novocure GmbH that includes: non-financial support, speaking and lecture fees, and travel reimbursement. Martin Misch reports a relationship with Servier that includes: speaking and lecture fees and travel reimbursement. David Wasilewski reports a relationship with German Cancer Consortium that includes: funding grants and non-financial support. David Wasilewski reports a relationship with Codman that includes: travel reimbursement. •If there are other authors, they declare that they have no known competing financial interests or personal relationships that could have appeared to influence the work reported in this paper.
