# Supplementary material for: Spinal leptomeningeal disease in patients with solid tumors: A single-center study on clinical presentation, diagnosis, treatment, and outcomes
Source: Brain Spine. 2026 Aug 29;6:106660. doi: 10.1016/j.bas.2026.106660 (PMC13629224; doi:10.1016/j.bas.2026.106660)
Supplement: Supplementary file 1 [file mmc1.docx]

**SUPPLEMENTARY DATA**

| **Supplementary Table 1: Survival data** | |  |  |
| --- | --- | --- | --- |
| **Sub-group** | **Patients (%)** | **Median OS (months) [95% CI]** | **p-value (log-rank)** |
| **Whole cohort** | 52 (100%) | 2.7 [2.0-3.7] |  |
| **Primary cancer type** |  |  | p=0.56 |
| Breast cancer | 16 (30.8) | 3.4 [1.5-NA] |  |
| Lung cancer (NSCLC) | 13 (25.0) | 2.9 [1.6-NA] |  |
| Malignant melanoma | 4 (7.7) | 1.1 [0.3-NA] |  |
| Other primary entities | 19 (36.5) | 2.5 [1.4-7.0] |  |
| **Age** |  |  | p=0.088 |
| <65 years | 36 (69.2) | 3.5 [2.4-6.8] |  |
| ≥65 years | 16 (30.8) | 1.6 [0.8-4.5] |  |
| **KPS** |  |  | p=0.42 |
| Bad KPS (<70%) | 26 (50.0) | 2.4 [1.4-7.0] |  |
| Good KPS (≥70%) | 26 (50.0) | 2.8 [2.0-4.6] |  |
| **Targetable mutations** |  |  | p=0.39 |
| Targetable mutation detected | 16 (30.8) | 3.4 [1.4-NA] |  |
| No targetable mutation detected | 36 (69.2) | 2.6 [1.5-4.5] |  |
| **Hydrocephalus in course of disease** |  |  | p=0.31 |
| Hydrocephalus | 5 (9.6) | 8.6 [1.3-NA] |  |
| No hydrocephalus | 47 (90.4) | 2.7 [2.0-3.7] |  |
| **Concurrent cranial and spinal LMD** |  |  | p=0.8 |
| Concurrent cranial and spinal LMD | 48 (92.3) | 2.6 [1.6-3.7] |  |
| Only spinal LMD | 4 (7.7) | 5.6 [0.5-NA] |  |
| **EANO-ESMO subtype** |  |  | p=0.081 |
| Type I | 25 (48.1) | 1.8 [1.2-3.7] |  |
| Type II | 27 (51.9) | 3.4 [2.5-7.0] |  |
| **MRI subtype** |  |  | p=0.55 |
| Type A | 24 (46.2) | 1.6 [1.2-3.7] |  |
| Type B | 6 (11.5) | 2.8 [2.5-NA] |  |
| Type C | 20 (38.5) | 3.6 [2.0-8.6] |  |
| Type D | 2 (3.8) | 4.9 [1.3-NA] |  |
| **Symptoms** |  |  |  |
| Cranial nerve deficits |  |  | p=0.0072 |
| Cranial nerve deficits at LMD diagnosis | 15 (28.8) | 1.4 [0.8-3.7] |  |
| No cranial nerve deficits at LMD diagnosis | 37 (71.2) | 3.5 [2.5-6.8] |  |
| Gait imbalance |  |  | p=0.022 |
| Gait imbalance at LMD diagnosis | 18 (34.6) | 1.4 [0.8-6.3] |  |
| No gait imbalance at LMD diagnosis | 34 (65.4) | 3.4 [2.5-6.8] |  |
| **Activity of primary disease at LMD** |  |  | p=0.93 |
| Active primary disease | 7 (13.5) | 3.4 [0.8-NA] |  |
| Inactive primary disease | 44 (84.6) | 2.7 [1.6-4.6] |  |
| **History of BrM before LMD diagnosis** |  |  | p=0.96 |
| History of BrM | 26 (50.0) | 2.5 [1.6-6.3] |  |
| No history of BrM | 26 (50.0) | 3.4 [1.4-6.9] |  |
| **Presence of BrM at LMD diagnosis** |  |  | p=0.49 |
| BrM at LMD diagnosis | 30 (57.7) | 2.5 [1.6-4.6] |  |
| No BrM at LMD diagnosis | 22 (42.3) | 3.3 [1.2-8.6] |  |
| **History of EcM before LMD diagnosis** |  |  | p=0.096 |
| History of EcM | 41 (78.8) | 3.4 [1.5-6.8] |  |
| No history of EcM | 11 (21.2) | 2.4 [2.0-NA] |  |
| **Presence of EcM at LMD diagnosis** |  |  | p=0.94 |
| EcM at LMD diagnosis | 36 (69.2) | 2.8 [1.5-4.5] |  |
| No EcM at LMD diagnosis | 16 (30.8) | 2.6 [2.0-NA] |  |
| **Vertebral metastasis at LMD diagnosis** |  |  | p=0.57 |
| Vertebral metastasis at LMD diagnosis | 26 (50.0) | 2.4 [1.4-6.9] |  |
| No vertebral metastasis at LMD diagnosis | 26 (50.0) | 2.9 [2.4-4.5] |  |
| **Intraspinal metastasis at LMD diagnosis** |  |  | p=0.48 |
| Intraspinal metastasis at LMD diagnosis | 10 (19.2) | 3.4 [1.6-NA] |  |
| No intraspinal metastasis at LMD diagnosis | 42 (80.8) | 2.6 [1.5-3.7] |  |
| **Pre-treatment before LMD diagnosis** |  |  | p=0.72 |
| Treatment-naïve | 45 (86.5) | 2.9 [0.8-NA] |  |
| Received pre-treatment | 7 (13.5) | 2.7 [2.0-4.5] |  |
| **Systemic therapy after LMD diagnosis** |  |  | p=0.0014 |
| Received systemic therapy after LMD diagnosis | 22 (42.3) | 4.6 [3.4-8.6] |  |
| No systemic therapy after LMD diagnosis | 30 (57.7) | 1.6 [1.2-2.9] |  |
| **TTx after LMD diagnosis** |  |  | p=0.04 |
| Received TTx after LMD diagnosis | 14 (27.0) | 6.8 [2.5-NA] |  |
| No TTx after LMD diagnosis | 38 (73.0) | 2.4 [1.4-3.5] |  |
| **Spinal intervention before or after LMD diagnosis** |  |  | p=0.054 |
| Underwent spinal intervention | 9 (17.3) | 7.0 [1.5-NA] |  |
| Did not undergo spinal intervention | 43 (82.7) | 2.6 [1.6-3.7] |  |

Summary of survival data including median overall survival (mOS), 95% confidence intervals, and statistical significance for selected patient and disease characteristics.

**Supplementary Figure 1: Representative MRI findings at LMD diagnosis**

**1B**

**1A**

Representative cranial and spinal MR images of a patient with classical LMD, i.e., MRI subtype A, as can be seen by the linear “sugar-coating”-like contrast-enhancement of the vestibulocochlear nerve and spinal cord (**1A**), and a patient with nodular LMD, i.e., MRI subtype B, consisting of extra-axial nodular contrast-enhancing lesions on cMRI and sMRI which have close contact with meninges or ependyma in the left side of the cerebellum and at multiple locations of the spinal cord (**1B**). Patients with both patterns at the time of LMD diagnosis were classified as “mixed” LMD, i.e., MRI subtype C.

**Supplementary Figure 2: Interindividual variability in disease progression**

A waterfall plot displaying patients’ course of disease divided into time between diagnosis of primary disease and LMD diagnosis, and LMD diagnosis to death (or date of last follow-up) in months. The median survival from diagnosis of primary disease to death is 32.7 months [24.9-44.3]. Additionally, it shows that the median time from diagnosis of primary disease to LMD diagnosis is 30.2 [18.9-42.6] and the median time from LMD diagnosis to death is 2.7 months [2.0-4.1]. The time between primary disease diagnosis and LMD diagnosis and the time between LMD diagnosis and death or last follow-up show a weak positive correlation (ρ=0.255).

**Supplementary Figure 3: Annual spinal leptomeningeal disease diagnoses at our center between 2011 and 2024**

A histogram overlayed with a regression plot depicting the number of sLMD diagnoses at our center annually between 2011 and 2024. The plot shows a trend indicating a statistically significant annual increase in LMD diagnoses by approximately 15.9%, with a coefficient for the diagnosis year of 0.16 (SE=0.043, z=3.73, p<0.001).

**Supplementary Figure 4: Trends in spinal leptomeningeal disease diagnosis**

**4A**

**4B**

**4D**

**4C**

Violin plots depicting the time intervals between diagnosis of primary disease and LMD diagnosis (**4A**), between diagnosis of parenchymal brain metastasis and LMD diagnosis (**4B**) and between cranial and spinal imaging used to ascertain the diagnosis of cranial and spinal LMD (**4C**). An alluvial plot visualizing the frequency and outcomes of lumbar punctures (LP) across multiple attempts (LP1, LP2, LP3). The diagram tracks the detection or absence of tumor cells at each LP attempt, illustrating the flow of patients and the changes in test results across successive LPs, thereby highlighting the diagnostic process over time. In total, 37 (71.2%) of the patients in our cohort have undergone at least one LP in the context of LMD diagnosis. In the first LP (LP1), 22 out of 37 patients (59.5%) tested positive. Of those, 16 didn’t undergo further LPs and 6 did (**D**).

**Supplementary Figure 5: Kaplan-Meier curves**

**5A**

**5B**

**5C**

**5D**

**5E**

**5F**

**5G**

Kaplan-Meier curves stratified by patients’ KPS status (i.e., ≤70% and >70%) showing that patients with KPS ≤70% have a mOS of 2.4 [95% CI: 1.4-7.0] in comparison to patients with KPS >70% who have a mOS of 2.8 months [2.0-4.6] (p=0.42) (**A**). Kaplan-Meier curves of the two sub-cohorts stratified by the presence of a targetable mutation; the sub-cohort of patients in whose primary tumor no targetable mutation was identified had a mOS of 2.6 months [1.5-4.5], whereas the mOS of the patients who have had a targetable mutation identified was 3.4 months [1.4-NA] (p=0.39) (**B**). Kaplan-Meier curves of the two sub-cohorts stratified by the presence of hydrocephalus. Surprisingly, the sub-cohort of patients who had hydrocephalus had a mOS of 8.6 months [1.3-NA] in comparison to the patients without hydrocephalus who had a mOS of 2.7 months [2.0-3.7] (p=0.31). This interesting difference might be due to the small sub-cohort of patients who have developed a hydrocephalus in the course of their disease (n=5) (**C**). Kaplan-Meier curves of the sub-cohorts stratified based on the activity of the primary disease at LMD diagnosis. Patients with an active primary tumor at LMD diagnosis had a mOS of 3.4 months [0.8-NA], whereas those who had an inactive tumor had a mOS of 2.7 months [1.6-4.6] (p=0.93) (**D**). Kaplan-Meier curves of the two sub-cohorts of the ESMO EANO subtype. Patients with type I LMD had a mOS of 1.8 months [1.2-3.7] as compared to patients with type II who had a mOS of 3.4 months [2.5-7.0] (p=0.081). Only patients who have received an LP were included in this analysis (**E**). Kaplan-Meier curves stratified by the LMD MRI subtype. Patients with type C LMD had the longest mOS with 3.6 months [2.0-8.6], followed by patients with type B LMD who had a mOS of 2.8 months [2.5-NA] and patients with type A with a notably shorter mOS of 1.6 months [1.2-6.3] (p=0.55) (**F**). Kaplan-Meier curves of the sub-cohorts stratified based on the status of systemic therapy before LMD; the sub-cohort of patients who received systemic therapy before LMD diagnosis had a mOS of 2.7 months [2.0-4.5], whereas those who didn’t had a mOS of 2.9 months [0.8-NA] (p=0.72) (**G**).
